# Triazine Derivative as Putative Candidate for the Reduction of Hormone-Positive Breast Tumor: *In Silico*, Pharmacological, and Toxicological Approach

**DOI:** 10.3389/fphar.2021.686614

**Published:** 2021-05-28

**Authors:** Muhammad Tayyab Imtiaz, Fareeha Anwar, Uzma Saleem, Bashir Ahmad, Sundas Hira, Yumna Mehmood, Manal Bashir, Saima Najam, Tariq Ismail

**Affiliations:** ^1^ Riphah Institute of Pharmaceutical Sciences, Riphah International University, Lahore, Pakistan; ^2^ Department of Pharmacology, Faculty of Pharmaceutical Sciences, Government College University, Faisalabad, Pakistan; ^3^ Faculty of Pharmacy, Lahore College of Pharmaceutical Sciences, Lahore, Pakistan; ^4^ Department of Pharmacy, COMSAT University, Abbottabad, Pakistan

**Keywords:** NMU, progesterone, estrogen, oxidative stress, inflammatory cytokines, breast tumor

## Abstract

**Background and objectives:** Breast cancer is a heterogeneous disease that poses the highest incidence of morbidity among women and presents many treatment challenges. In search of novel breast cancer therapies, several triazine derivatives have been developed for their potential chemotherapeutic activity. This study aims to evaluate the N-nitroso-N-methyl urea (NMU)–induced anti–mammary gland tumor activity of 2,4,6 (O-nitrophenyl amino) 1,3,5-triazine (O-NPAT).

**Methods:** The *in silico* modeling and *in vitro* cytotoxicity assay were performed to strengthen the research hypothesis. For *in vivo* experimentation, 30 female rats were divided into five groups. Group I (normal control) received normal saline. Group II (disease control) received NMU (50 mg/kg). Group III (standard control) was treated with tamoxifen (5 mg/kg). Groups IV and V received O-NPAT at a dose level of 30 and 60 mg/kg, respectively. For tumor induction, 3 intraperitoneal doses of NMU were given at a 3-week interval, whereas all treatment compounds were administered orally for 14 consecutive days. Biochemical and oxidative stress markers were estimated for all experimental animals. DNA strand breakage alongside inflammatory markers was also measured for the analysis of inflammation. The hormonal profile of progesterone and estrogen was also estimated.

**Results:** The test compound presented a significant reduction in organ weight and restored the hepatic and renal enzymes. O-NPAT treatments enhanced the antioxidant enzyme level of catalase (CAT), superoxide dismutase (SOD), and total sulfhydryl (TSH), with a highly significant reduction in lactate dehydrogenase (LDH) and lipid peroxidation. Also, the decrease in fragmented DNA, hormonal levels (estradiol and progesterone), and inflammatory cytokines (IL-6 and TNF-α) justified the dosage efficacy further supported by histopathological findings.

**Conclusion:** All results indicated the anti–breast tumor activity of O-NPAT and presented its possibility of exploitation for beneficial effects in breast cancer treatment.

## Introduction

“Cancer” cites more than 100 sorts of diseases and is one of the leading public health concerns and global health hazards (Siegel et al., 2019). It encompasses a diverse population of abnormal cells that are genetically modified to overcome cell proliferation barriers and able to metastasize other body tissues (Harbeck et al., 2019). Among varied cancer types, breast cancer is the most prevailing neoplastic disease and the leading cause of death among women worldwide (Yankuzo et al., 2018). This malignancy is responsible for more than 1.5 million diagnoses and five hundred thousand deaths annually worldwide, the incidence of which is expected to reach up to 3.2 million cases per year by 2050 (Abolghasemi et al., 2020).

Breast cancer is a heterogeneous disease that is an aggregate of diverse histological features, assorted medical prognoses, and varied clinical responses (Assi et al., 2013). From a clinical perspective, it is classified into three major subtypes, that is, hormone receptor–positive tumor (HR+) (either estrogen receptor–positive (ER+) or progesterone receptor–positive (PR+) tumors), human epidermal growth factor receptor 2 overexpression (HER2+), and triple-negative breast cancer (TNBC) (Nagini, 2017). The genesis of breast tumor cells is a complex multistage process and involves the interaction of several risk factors that participate in tumor initiation and disease progression (Sun et al., 2017). The principal causes of this are aging, family history, genetic susceptibility, hormonal alterations, viral or bacterial infections, exposure to electromagnetic radiations, xenoestrogens, and oxidative stress (Huo et al., 2014; Atoum et al., 2020). Risk factors also include lifestyle modifications such as obesity, unhealthy way of living, physical inactivity, smoking, and use of alcohol (Thomson et al., 2014).

The role of female hormones including estrogen and progesterone is crucial for the comprehension of breast development, understanding metastatic pathologies, planning interventions, and avoiding treatment complications (Jesinger and Radiology, 2014). Estrogen, the most important of all, helps in the synthesis of an extensive breast ductal network while also being involved in tumor initiation and disease progression. It acts on breast epithelial cells, making them multiply faster with increased chances of mutant cancerous cell production (Samavat and Kurzur, 2015). Other pathologic factors include the increased levels of inflammatory cytokines such as interleukins (IL-6 and IL-1β) and tumor necrosis factor (TNF-α), both of which contribute to tissue inflammation, tumor invasiveness, and less response to endocrine therapies (Goldberg and Schwertfeger, 2010). Also, oxidative stress participates in tumor pathology by altering the genetic stability and causing DNA damage, oncogene mutations, cell proliferation, and carcinogenesis and further complicating the disease state (Hecht et al., 2016).

Regarding the treatment procedures, there have been remarkable advances in breast cancer awareness, diagnosis, etiology, and therapeutics, which have helped decrease the overall rate of cancer-related deaths worldwide (Shapiro, 2018). Common treatment regimens consist solely of or combinations of endocrine therapy, chemotherapy, radiotherapy, and surgery (Sledge et al., 2014). Early-stage breast tumors are mostly treated with endocrine therapy and breast-conserving surgery (BCS), whereas the stage III and IV tumor states are aided by mastectomy and adjuvant chemotherapy, whereas radiation therapy is usually performed to evade post-surgery tumoral recurrence (Miller et al., 2019).

Despite such disease refinements, there still exist many challenges that ought to be addressed such as patient safety profiles, endocrine therapy resistance, cancer relapse, and identification of new molecular targets (Pellegrino et al., 2018). For this purpose, several triazine-based compounds have been investigated for their chemopreventive properties (Srivastava et al., 2017). Several reports have shown the potential of 1,3,5-triazine as having cardiotonic, neuroleptic, antihistamine, tuberculostatic, anti-HIV, antiviral, and significant anticancer properties (Cascioferro et al., 2017). Among chemotherapeutic triazine-based compounds, altretamine, azacitidine, and decitabine are currently being employed as antineoplastic agents for treating ovarian cancer, chronic myeloid leukemia, and acute myeloid leukemia, respectively (Moreno et al., 2018). Various substitutions on the 1,3,5-triazine ring have to yield medicinal compounds with a pronounced effect on cell proliferation. It acts by the inhibition of an epidermal growth factor receptor (EGFR) *via* substrate phosphorylation and resultant receptor inactivity (Yan et al., 2018). Among other substituents, 1,3,5-triazine hydrazones have demonstrated antiproliferative activity against colon and breast carcinoma (H AI Rasheed et al., 2020). Imidazolyl 1,3,5-triazines have proven aromatase inhibitory activity and might find their application in ER+ tumors (Singla et al., 2015). The series of hydrazone and fluoro triazine compounds have also been reported as a particular blocker of EGFR (H AI Rasheed et al., 2020). The test substance, 2,4,6 (O-nitrophenyl amino) 1,3,5 triazine (O-NPAT), also belongs to this novel class of drugs, the knowledge for which is grounded on its novice status, and no scientific data are existing to our utmost knowledge. Also, due to the ever-increasing cancer burden, there has always been room for the investigation of new medicinal compounds with desired characteristics. It is therefore considered worthwhile to inquire into the anti–mammary gland tumor activity of O-NPAT *via in silico*, *in vitro*, and *in vivo* study models. The purpose of this study is to evidence the antitumor potential of 2,4,6 (O-nitrophenyl amino) 1,3,5 triazine (O-NPAT) against hormone-positive (HR+) mammary gland tumors in female rats.

## Material and Methods

### Drugs and Chemicals

Carboxymethylcellulose (CMC), formalin, isoflurane, hydrogen peroxide (H_2_O_2_), trichloroacetic acid (TCA), pyrogallol solution, thiobarbituric acid (TBA), O-dianisidine, 5, 5-dithiobis-2-nitrobenzoic acid (DNTB), diphenylamine solution, ethylene diamine tetra acetic acid (tris-EDTA), hydrochloric acid (HCl), and sulfuric acid (H_2_SO_4_) were of analytical grade and purchased from the local vendor that deals with Merck Germany. N-Nitroso-N-methyl urea (NMU) was used as a tumor-inducing agent (Oakwood Chemicals, United States). Tamoxifen was employed as a standard anticancer agent (Nolvadex, AstraZeneca). ELISA kits for the quantitation of estradiol E2 and progesterone were purchased from Perkin Elmar Health Sciences, Inc., United States. Meanwhile, interleukin (IL-6) and tumor necrosis factor-alpha (TNF-α) ELISA kits were purchased from Abcam, United States.

### *In Silico* Modeling

Receptor-binding properties for O-NPAT were compared with the standard: tamoxifen designed at estrogen receptor-alpha (ERα), estrogen receptor-beta (ERβ), progesterone receptor (PR), and epidermal growth factor receptor (EGFR). The 3D crystal structure of all receptors was retrieved from the RCSB Protein Data Bank, and docking studies of various nuclear receptors were computed using AutoDock tool (version 1.5.6.), which explores the binding sites of a docked molecule utilizing the Lamarckian genetic algorithm (LGA). Ten docked conformations were obtained after protein–ligand docking at the nuclear receptors and tyrosine kinase receptor, where the optimal conformations were screened in terms of lowest binding energy among several binding interactions. Cluster analysis of protein binding sites with the lowest binding energy was further explored using PyMol Molecular Graphics System offline software, Protein-Ligand Interacting Profile (PLIP), and Protein Plus online software.

### *In Vitro* Cytotoxicity Assay

The MCF-7 breast cancer cell line was employed for *in vitro* characterization of a test compound. Following the standard procedure (Abu-Dahab and Afifi, 2007), MCF-7 cells were first cultured to be seeded enough to present reliable results. The cells were rinsed with PBS; 10 ml of supplement medium were added to them, and they were centrifuged at 3,500 rpm for 10 min to make a uniform suspension. Then 100 μl of suspension was placed in a well plate and incubated for 24 h at 37°C. After culture preparation, O-NPAT was first dissolved in a vehicle (0.5% CMC), diluted in a medium, and added to respective micro-wells alongside standard doxorubicin and control (blank), and incubation was done for 72 h. At the end, all the wells were analyzed for possible cell growth *via* dimethylthiazol diphenyl tetrazolium (MTT) assay, and the IC50 value was calculated using WinNonlin Professional software (version 5.0.1).

#### MTT Assay

The working solution containing 10 µl of MTT solution (500 mg of MTT in 10 ml PBS) was added to each well and incubated for 4 h at 37°C. The purple-colored formazan crystals were formed, which were solubilized by the addition of 50 µl DMSO and incubated at 37°C for 30 min. The absorbance was recorded at 540 nm, and percentage viable cells were calculated (Bahuguna et al., 2017).

$$\mathrm{\% growth inhibition=}\frac{\mathrm{100-Mean O}\mathrm{.D}\mathrm{. of test compound}}{\mathrm{Mean O}\mathrm{.D}\mathrm{. of control}}\mathrm{\times 100}$$ (1)

### *In Vivo* Antitumor Activity

#### Experimental Animals

A total of 30 adult female Wistar rats weighing 120–140 g were obtained from Riphah International University, Lahore. All female rats were housed in stainless steel cages (not more than six animals per cage) in a temperature-controlled environment (22 ± 2°C) with a relative humidity of 45 ± 5% with natural light and dark cycles. The animals were provided with a rodent’s pellet diet and excess water free from chemical contamination. The permission for the study was obtained from the Research Ethical Committee of Riphah International University under the authorized number of REC/RIPSLHR/2017/042.

#### Experimental Protocol

The animals were divided into five groups, each containing six female rats (*n =* 6). Group I (normal control) received normal saline (10 ml/kg) intraperitoneally. Group II (disease control) received freshly prepared N-nitro N-methyl urea (NMU) (50 mg/kg) intraperitoneally. For mammary gland tumor induction, the NMU dose was repeated every 3 weeks for a total of three times. Group III (standard control) was treated with tamoxifen (5 mg/kg). Groups IV and V were administered with the test substance: O-NPAT at the dose of 30 and 60 mg/kg, respectively. After 12 weeks of tumor induction, the treatment groups (Groups III–V) received their respective treatment orally for 15 consecutive days.

#### Serum Collection and Tissue Preparation

Twenty-four hours after the last dose, all female rats were anesthetized using isoflurane, and serum preparation was carried out by collecting the blood *via* cardiac puncture before euthanizing the rats. The blood was centrifuged at 4,000 rpm for 20 min to obtain a clear supernatant that was used for the estimation of liver and renal functioning. The rats were then euthanized *via* cervical dislocation, where the right and left inguinal mammary glands were removed by gently peeling the animal’s skin sideways, weighed, and preserved for biochemical and histological analysis.

#### Preparation of Mammary Tissue Homogenate

Following the dissection of all experimental animals, mammary glands were removed and weighed, and 10 ml of phosphate buffer (pH 7.4) was added for tissue homogenate preparation. Tissues were first standardized using a homogenizer and then centrifuged at 4,000 rpm for 30 min. A fat layer was found overlying the supernatant that was carefully removed, and inferior tissue product was used for the analysis of various enzyme activities.

### Biochemical Assay of Enzyme Levels

#### Measurement of Total Protein Level

Following the standard procedure (Lowry et al., 1951), 4.5 ml of reagent 1 [2% sodium carbonate (Na₂CO₃) in 0.1 N sodium hydroxide (NaOH), 1% sodium potassium tartrate, and 0.5% copper sulfate (CuSO_4_)] and 0.2 ml of tissue homogenate were mixed and incubated for 10 min at 37°C. Then 0.5 ml of reagent 2 (2 N Folin phenol) was added, and again, incubation was done for 30 min at 37°C. Absorbance was then recorded using a UV–visible spectrophotometer at 660 nm.

#### Assay for Catalase Levels

The catalase activity was estimated as a result of the decomposition of hydrogen peroxide (H_2_O_2_) into oxygen and water (Hira et al., 2019). In brief, 1.95 ml phosphate buffer (pH 7.4), 0.05 ml tissue supernatant, and 1 ml 30 mM hydrogen peroxide were mixed in a stepwise manner, and absorbance was recorded at 240 nm using a UV–visible spectrophotometer.

#### Assay for Superoxide Dismutase Levels

SOD was determined according to Kim et al. (2016). The assay mixture was prepared by the addition of 0.1 ml mammary tissue homogenate (10% w/v), 0.1 ml of pyrogallol solution (pH 7.4), and 2.8 ml of potassium phosphate buffer (pH 7.4) in a test tube. The absorbance of the mixture was measured at 325 nm using a UV–visible spectrophotometer (Shamadzo, Japan).

#### Assay of Nitrite Activity

Griess reagent was used for an indirect estimation of the nitrite level (Bais et al., 2015), where 1 ml each of tissue supernatant and Griess reagent (0.1% naphthyl ethylenediamine dihydrochloride and 1% sulfanilamide in 2.5% phosphoric acid) were added in a test tube and incubated for 15 min at 37°C. Absorbance was then recorded at 546 nm spectrophotometrically.

#### Determination of Reduced Glutathione

In a test tube, 1 ml of mammary gland homogenate and 1 ml of 10% trichloroacetic acid were added. Then 4 ml of phosphate buffer solution (pH 7.4) and 0.5 ml of DTNB reagent (5,5-dithiobis-2-nitrobenzoic acid) were added stepwise to the aliquot of the supernatant. Absorbance was measured using a UV–visible spectrophotometer at 412 nm (Hira et al., 2019).

#### Determination of Total Sulfhydryl

TSH levels were assayed as per the method of Adefisan et al. (2019). The reaction mixture was prepared by adding 1 ml each of mammary gland homogenate and 5,5′-dithiobis 2-nitrobenzoic acid (DTNB). A chromosphere product was formed, the absorbance of which was recorded at 412 nm using a UV–visible spectrophotometer.

#### Determination of Malondialdehyde Levels

The reaction mixture contained 3 ml of thiobarbituric acid (TBA) reagent (15% w/v TCA, 0.38% w/v TBA, and 0.25M HCl) and 1 ml of tissue supernatant, mixed well, and kept in a water bath for 30 min at 70°C. Later, this solution was cooled for 15 min in an ice bath and centrifuged at 4,000 rpm for 10 min. The clearly defined upper layer was removed, the absorbance of which was recorded at 523 nm (Draper et al., 1993).

#### Determination of Lactate Dehydrogenase Levels

LDH estimation was performed according to Adefisan et al. (2019). In a test tube, 1 ml of pyruvate solution, 1 ml of phosphate buffer, and 0.04 ml homogenate sample were added. The mixture was mixed thoroughly, and the changes in absorbance were monitored at 340 nm for 3 min with 1-min intervals in between using a UV–visible spectrophotometer.

### Percentage DNA Fragmentation

Following the standard procedure (Adefisan et al., 2019), mammary gland tissues were first homogenized in 10 volumes of Tris-EDTA buffer (pH 8.0) and centrifuged at 4,000 rpm for 30 min to obtain the intact chromatin pellet (A) separated from the fragmented supernatant (B) and were suspended in Tris-EDTA buffer. Then 0.5 ml aliquots each with pellet A and supernatant B were taken in respective test tubes, and 1.5 ml of freshly prepared diphenylamine solution (0.5% diphenylamine dissolved in 90% sulfuric acid) was added, followed by incubation for 20 h at 37°C. Absorbance was recorded at 620 nm, and percentage of fragmented DNA was calculated using the following formula:

$$\mathrm{\%fragmented DNA=}\frac{\mathrm{Abs}\mathrm{.of supernatant (B)}}{\mathrm{Abs}\mathrm{.of pellet (A)+Abs}\mathrm{.of supernatant (B)}}\mathrm{\times 100}.$$ (2)

### Enzyme-Linked Immunosorbent Assay

ELISA tests for the quantitation of estradiol (E2), progesterone (PR), interleukin (IL-6), and tumor necrosis factor-alpha (TNF-α) were performed. In brief, the well plate was coated with antigen (serum sample), and primary antibody was added, followed by the addition of secondary enzyme-conjugated antibody. The reaction was ceased by adding the stop solution, and absorption was recorded using a microplate reader (Diamate Bio-Tech., United Kingdom).

### Histopathological Studies

The female rats’ inguinal mammary glands, liver, and kidneys were removed and fixed in paraffin wax, sliced into 3- to 4-μm longitudinal sections, and stained in eosin and hematoxylin dye. The slides were examined under a light microscope, and the histoarchitecture was studied.

### Statistical Analysis

The data were represented as mean ± SEM. The resultant values were evaluated using one-way analysis of variance (ANOVA) with subsequent post hoc Dunnett’s test, where the level of significance was considered at *p* < 0.05, *p* < 0.01, and *p* < 0.001, respectively, using GraphPad Prism (version 5.01).

## Results

### Receptor–Ligand Binding Affinities

**FIGURE 1:**
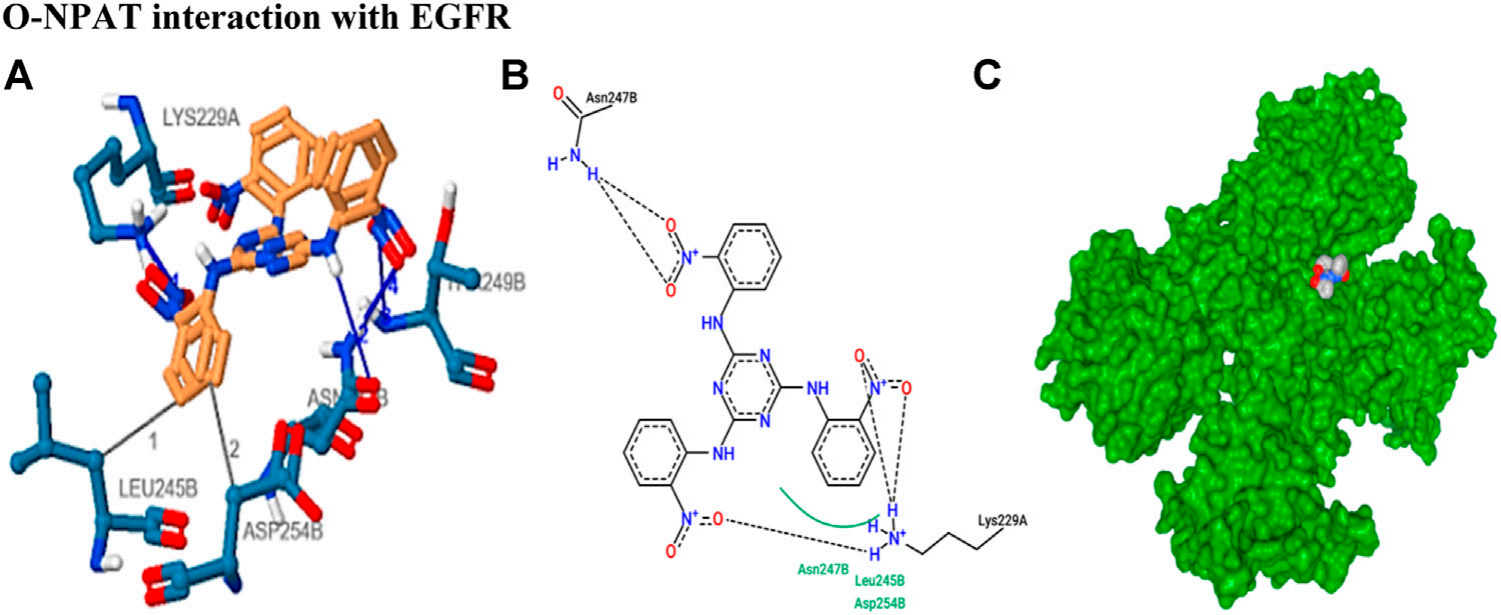
O-NPAT interaction with EGFR. **(A)** Blue color residues show receptor interaction with orange color ligand. **(B)** The ligand–receptor interaction is stabilized by three hydrogen bonds and hydrophobic interactions. **(C)** Tyrosine kinase receptor (green) in coordination with ligand (O-NPAT) shown as a gray, blue, and red sphere.

**FIGURE 2:**
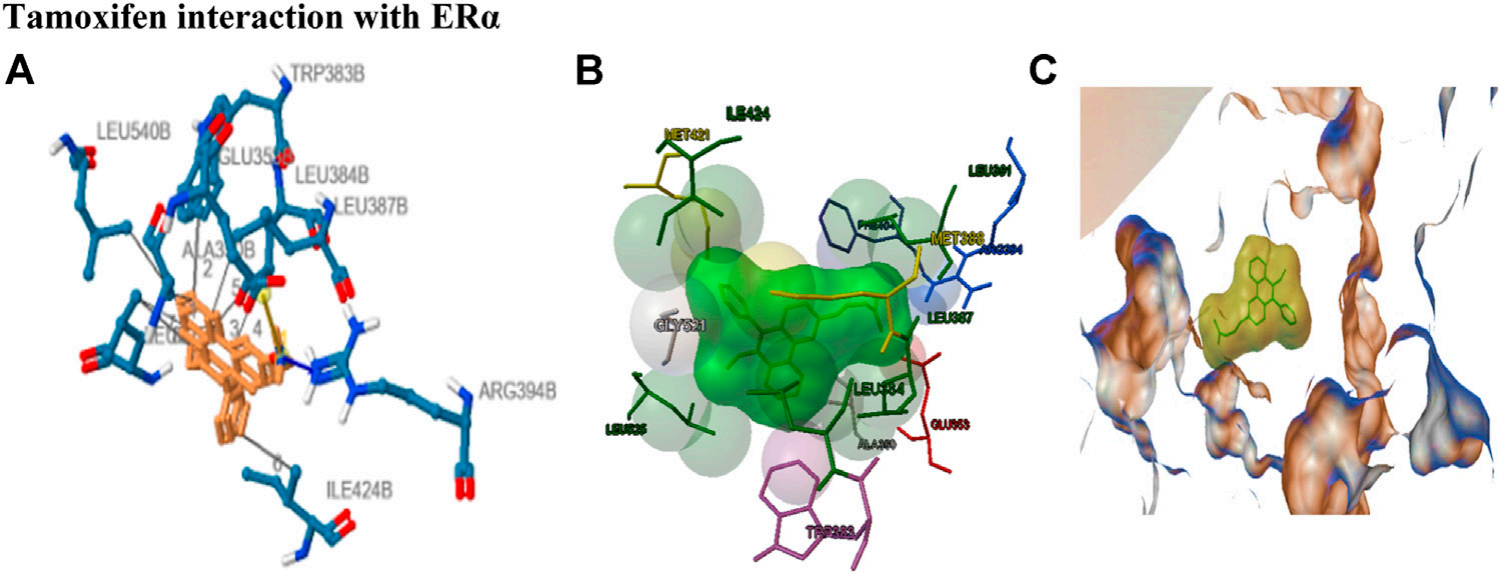
Tamoxifen interaction with ERα receptor. **(A)** Blue color residues show receptor interaction with orange color ligand. **(B)** The best ligand–receptor interaction is modeled by the auto dock. **(C)** Tamoxifen is shown as a molecular sphere inside the binding pocket of the receptor.

The stability of drug docking describes how well the drug has interacted with various protein receptors (EGFR, ERα, ERβ, and PR). O-NPAT (binding energy = −7.1 kcal/mol) showed good interaction at EGFR in comparison to tamoxifen (binding energy = −7.7 kcal/mol); on the other hand, O-NPAT showed great deviation at ERα, ERβ, and PR in comparison to tamoxifen. The main interacting residues of O-NPAT with EGFR were LYS229, LEU245, ASN247, THR249, and ASP254, where the best-docked confirmation of O-NPAT at EGFR showed three hydrogen bonds. In the case of tamoxifen, the main interacting residues were ASP279, HIS280, LEU243, and MET244 (Figures 1, 2).

### *In Vitro* Cytotoxicity Assay

The well plate had a cell count of 5 × 10^3^ cells per well, where cell viability was found to be 4,250 cells/well. The results showed a 15% inhibition of adenocarcinoma cells (Table 1) at a concentration of 30 μm, demonstrating the O-NPAT cytotoxic activity and its practicality for animal studies.

**TABLE 1:** *In vitro* cytotoxicity assay.

Test compound	Concentration (μm)	Percentage inhibition
2,4,6 (O-nitrophenyl amino) 1,3,5-triazine replaced as (O-NPAT)	30	15 ± 1.5
Doxorubicin (standard)	30	85.2 ± 2.5

Data are represented as mean ± SEM, *n* = 3.

### Effect of O-NPAT Treatments on Animal’s Body Weight

**FIGURE 3:**
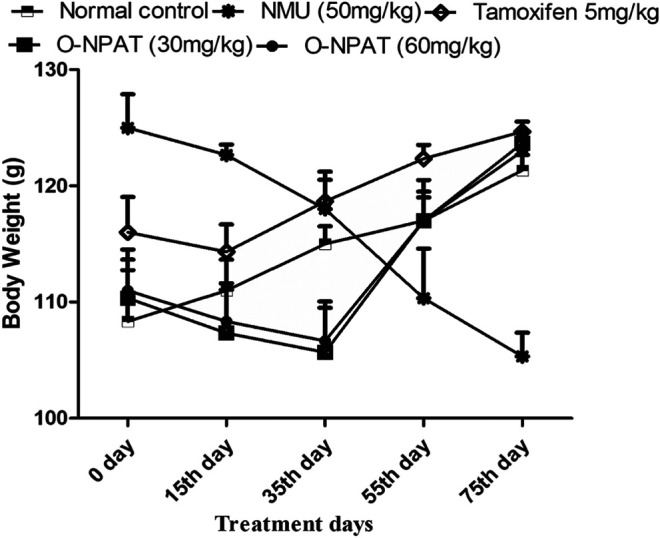
Effect of O-NPAT treatments on animal’s body weight. Data are represented as mean ± SEM, *n* = 6.

A significant decrease (*p* < 0.05) in the body weight of animals after 50 mg/kg NMU administration showed the development of breast tumor and the morbidity associated with it. A highly significant (*p* < 0.001) increase after O-NPAT treatments showed the test compound’s competence in the eradication of breast tumors (Figure 3).

### Effect of O-NPAT Treatments on the Weight of Mammary Glands

**FIGURE 4:**
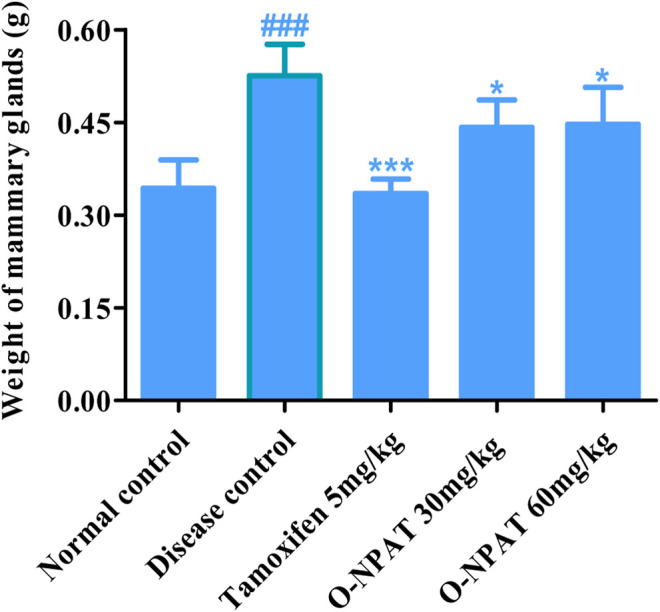
Effect of O-NPAT treatments on the weight of mammary glands. Data are represented as mean ± SEM, *n* = 6, ###*p* < 0.001 and ***p < 0.001, *p < 0.05 as compared with normal control and diseased group, respectively.

A highly significant increase (*p* < 0.001) in the mammary gland weight of the animals treated with 50 mg/kg NMU indicated the progression of breast carcinoma (0.52 ± 0.021g). O-NPAT (30 and 60 mg/kg) displayed a significant (*p* < 0.05) reduction in the organ weight when compared with the disease group (Figure 4).

### Effect of O-NPAT Treatments on LFTs and RFTs

The levels of liver functioning parameters (ALT, AST, and ALP) and renal functioning parameters (urea and creatinine) were quantified in the serum samples of all experimental group animals. These enzymes and by-products were metabolized and released *via* the liver and renal routes and may indicate the organ’s toxicity. The results showed a significant increase in the liver and kidney functioning parameters in the disease control group when compared with the normal control group. Treatment with O-NPAT at both dose levels significantly reduced the indicating parameters of liver and kidney functioning (Table 2).

**TABLE 2:** Effect of O-NPAT treatments on LFTs and RFTs.

Experimental group	ALT (U/L)	AST (U/L)	ALP (U/L)	Urea (mg/dl)	Creatinine (mg/dl)
Normal control	62.60 ± 1.5	76.000 ± 2.5	186.0 ± 2.921	39.01 ± 2.63	0.900 ± 0.037
Disease control	113.83 ± 2.8^a^	94.00 ± 2.782^b^	240.8 ± 2.3^a^	45.83 ± 1.53	0.700 ± 0.037^b^
Tamoxifen 5 mg/kg	90.83 ± 2.6^c^	54.0 ± 2.7^c^	199.6 ± 3.42^d^	44.01 ± 1.46	0.817 ± 0.031
O-NPAT 30 mg/kg	36.83 ± 2.1^c^	67.5 ± 7.1^d^	177.1 ± 3.8^c^	38.05 ± 1.2^e^	0.842 ± 0.037
O-NPAT 60 mg/kg	54.6 ± 3.1^c^	70.83 ± 8.5^e^	168.1 ± 16.5^c^	38.60 ± 2.6	0.817 ± 0.054

Data are represented as mean ± SEM (*n* = 6).

^a^ *p* < 0.001.

^b^ *p* < 0.05 in comparison to normal control group.

^c^ *p* < 0.001.

^d^ *p* < 0.01.

^e^ *p* < 0.05 in comparison to disease control group.

### Effect of O-NPAT Treatments on Oxidative Stress Biomarkers

#### Effect on Total Protein and Catalase Levels

**FIGURE 5:**
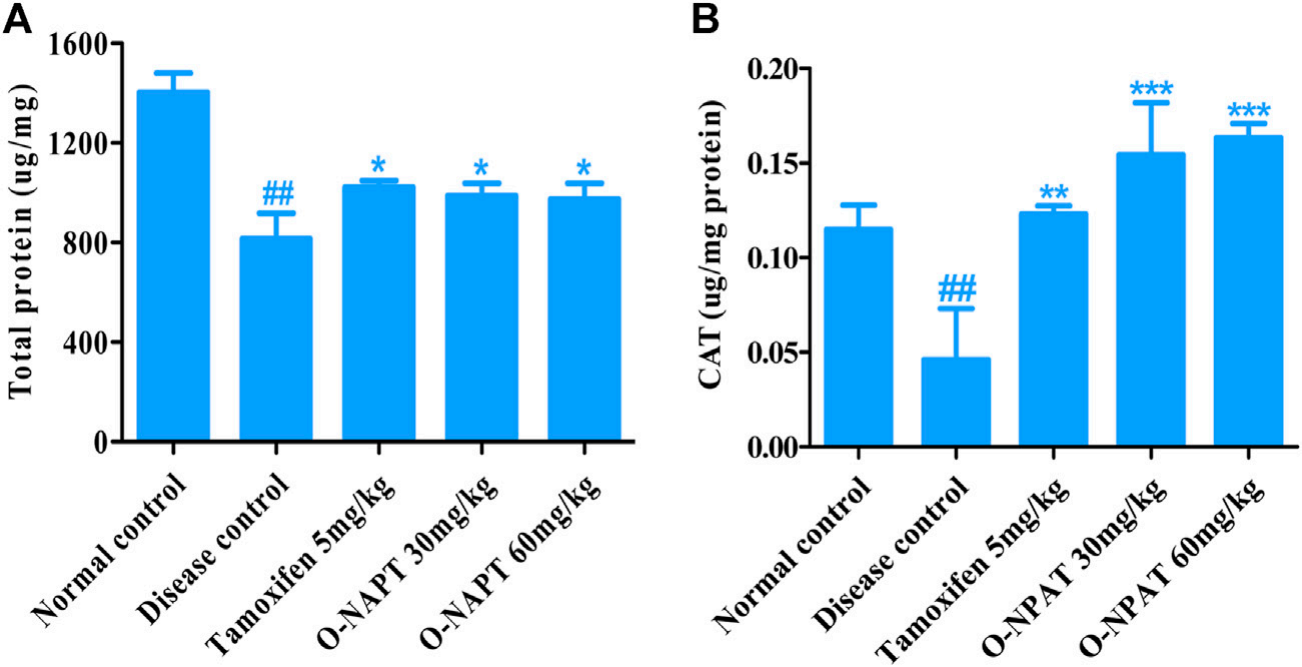
Effect of O-NPAT treatments in rats treated with NMU. **(A)** Total protein levels. **(B)** Catalase (CAT) levels. Data are represented as mean ± SEM, n = 3, ##*p* < 0.01 and ***p < 0.001, **p < 0.01, *p < 0.05 as compared with normal control and diseased group, respectively.

The total protein levels in body cells are a worthy indicant of drug toxicity and treatment competence. O-NPAT at 30 and 60 mg/kg dose levels ad tamoxifen (5 mg/kg) showed a comparable significant increase (*p* < 0.05) in total protein content in comparison to the disease control group (Figure 5A). Meanwhile, catalase (CAT) is an antioxidant enzyme which decomposes hydrogen peroxide (H_2_O_2_) into oxygen and water, thus preventing the ROS-generated cellular damage (Nandi et al., 2019). O-NPAT at doses of 30 and 60 mg/kg showed an almost parallel highly significant increase (*p* < 0.001) in CAT levels in contrast to the disease control group (Figure 5B).

#### Effect of O-NPAT Treatments on SOD, GSH, TSH, and Nitrite Levels

**FIGURE 6:**
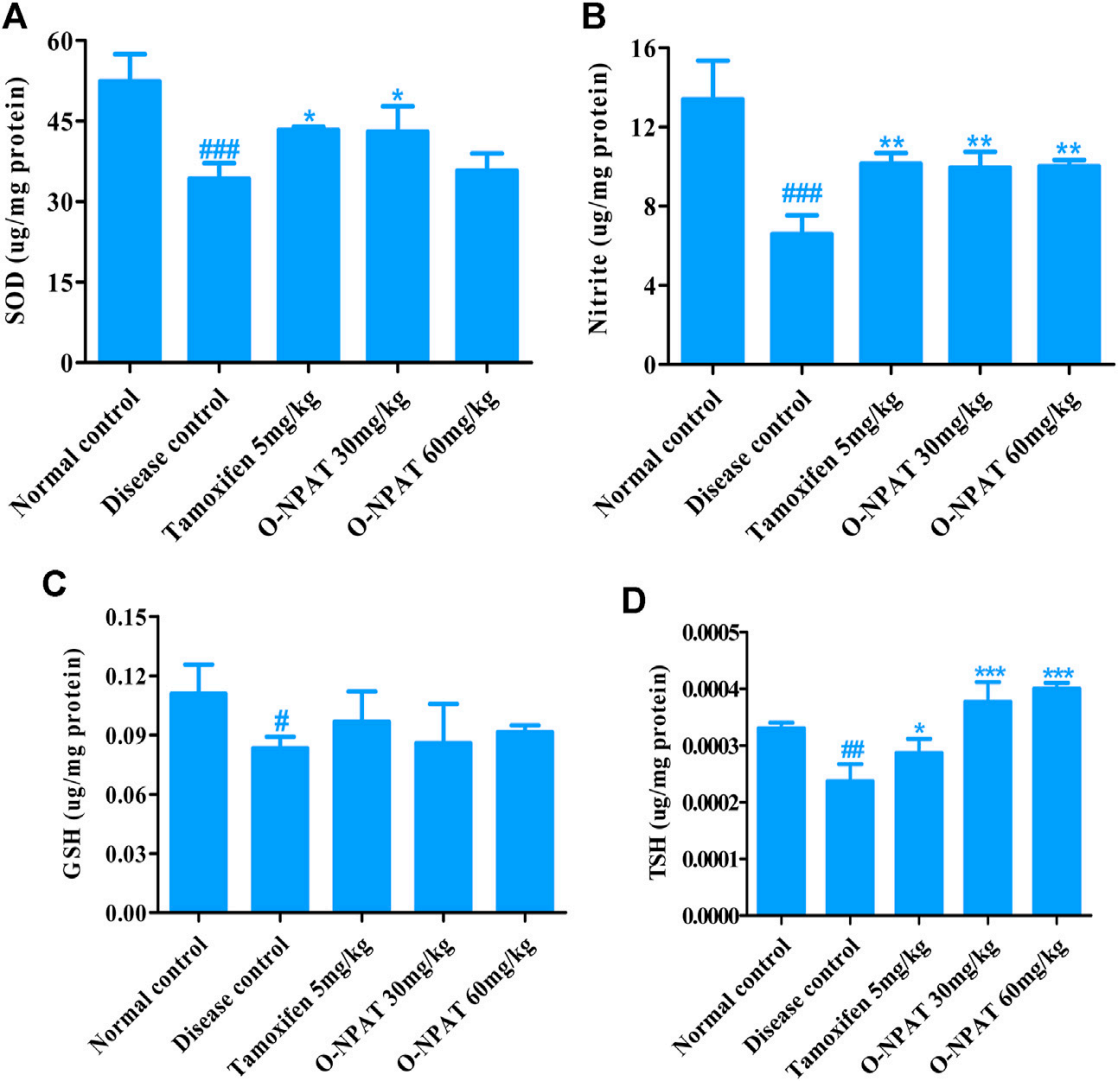
Effect of O-NPAT treatments on different enzyme levels in rats treated with NMU. **(A)** Superoxide dismutase (SOD) levels. **(B)** Nitrite or nitric oxide (NO) level. **(C)** Reduced glutathione (GSH) level. **(D)** Total sulfhydryl (TSH) level. Data are represented as mean ± SEM (*n* = 3) where, ###*p* < 0.001, ##*p* < 0.01 #*p* < 0.05 and ***p < 0.001, **p < 0.01, *p < 0.05 shows comparison with normal control and diseased group, respectively.

The SOD, GSH, and TSH enzymes are integral components of normal body cell functioning and also play an imperative role in cellular toxicity and disease conditions and therefore needed to be monitored. The superoxide dismutase (SOD) enzyme catalyzes the superoxide’s dismutation into either molecular oxygen (O_2_) or hydrogen peroxide (H_2_O_2_) and helps prevent tissue damage. Tamoxifen (5 mg/kg) and O-NPAT (30 mg/kg) showed an almost equivalent significant increase (*p* < 0.05) in SOD enzyme levels as compared to NMU (50 mg/kg) (Figure 6A). Nitrite or nitric oxide (NO) is a signaling molecule that participates in immune response, vasodilatation, and many other physiological processes and is therefore an imperative component of cancer mechanisms. In the case of nitrite levels, tamoxifen (5 mg/kg) and O-NPAT at both doses of 30 and 60 mg/kg exhibited almost parallel moderate significant increase (*p* < 0.01) in the nitrite level as compared to disease control animals (Figure 6B). Reduced glutathione (GSH) also serves as a neurotransmitter and regulates the different biochemical reactions (Pompella et al., 2003). All treatment groups including tamoxifen (5 mg/kg) and O-NPAT (30 and 60 mg/kg) showed nonsignificant deviation in GSH enzyme levels in comparison to the disease control group (Figure 6C). Sulfhydryls are the hydroxyl sulfur analogs that contribute to protein tertiary structure formation, protein stability, enzyme activation, and metal chelation (Winther and Thorpe, 2014). The results of the TSH levels showed that O-NPAT at 30 and 60 mg/kg dose levels significantly (*p* < 0.001) increases the levels of TSH when compared with the disease control group, which was also more significant than the standard control group (Figure 6D).

#### Effect of O-NPAT Treatments on Disease Biomarkers

**FIGURE 7:**
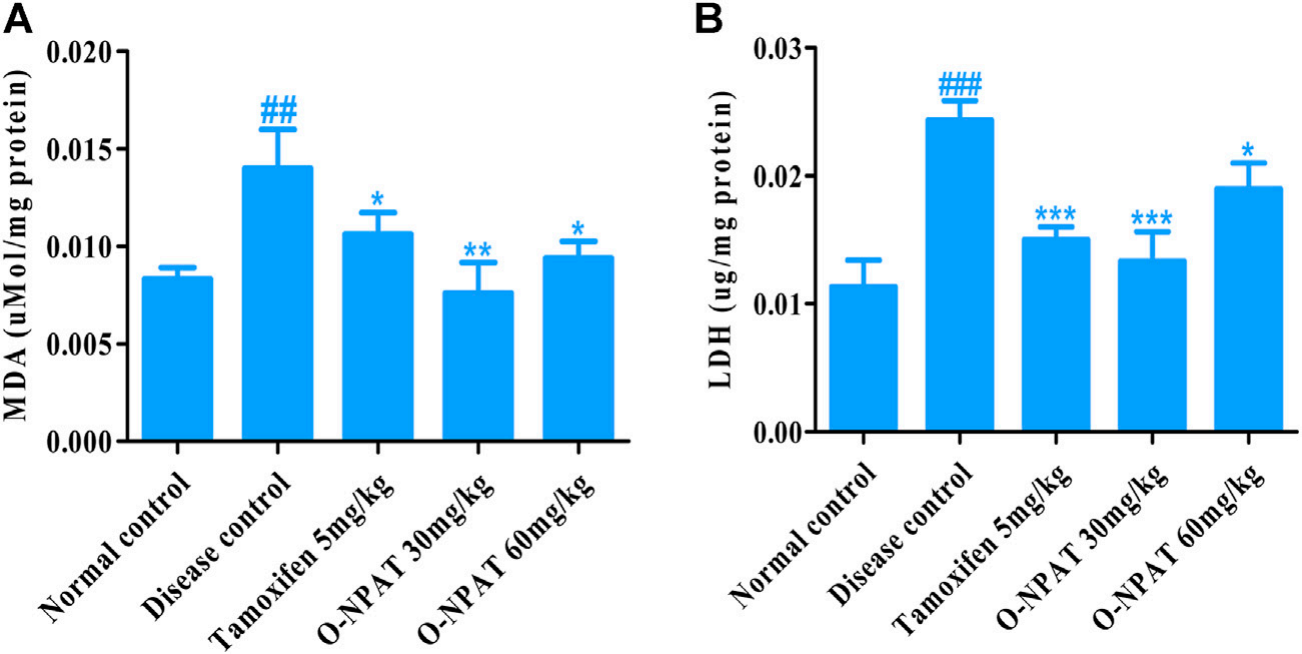
Effect of O-NPAT treatments on disease biomarkers in rats treated with NMU. **(A)** Malondialdehyde (MDA) level. **(B)** Lactate dehydrogenase (LDH) level. Data are represented as mean ± SEM, *n* = 3, ###*p* < 0.001, ##*p* < 0.01 and ***p < 0.001, **p < 0.01, *p < 0.05 as compared with normal control and disease control group, respectively.

Indicators of the level of cellular toxicity such as malondialdehyde (MDA) and lactate dehydrogenase (LDH) were monitored for all experimental group animals. These by-products are associated with several disease conditions and therefore needed to be monitored. Malondialdehyde is a by-product of polyunsaturated fatty acid degradation and produces toxic cellular stress while also acting as a mutagen. The results showed O-NPAT (30 mg/kg) treatment exhibiting a significant decline in the levels of MDA in comparison to the disease control. O-NPAT (60 mg/kg) and tamoxifen also showed an equivalent decrease in lipid peroxidation levels when compared with the disease control (Figure 7A). Meanwhile, lactate dehydrogenase (LDH) is an enzyme released during tissue injury, and it also acts as a tumor marker. Tamoxifen (5 mg/kg) and O-NPAT (30 mg/kg) exhibited a parallel decrease in the levels of LDH when compared to the normal control group, that is, a highly significant reduction (*p* < 0.001) in contrast to the disease control animals (Figure 7B).

### Effect of O-NPAT Treatments on Percentage DNA Fragmentation

**FIGURE 8:**
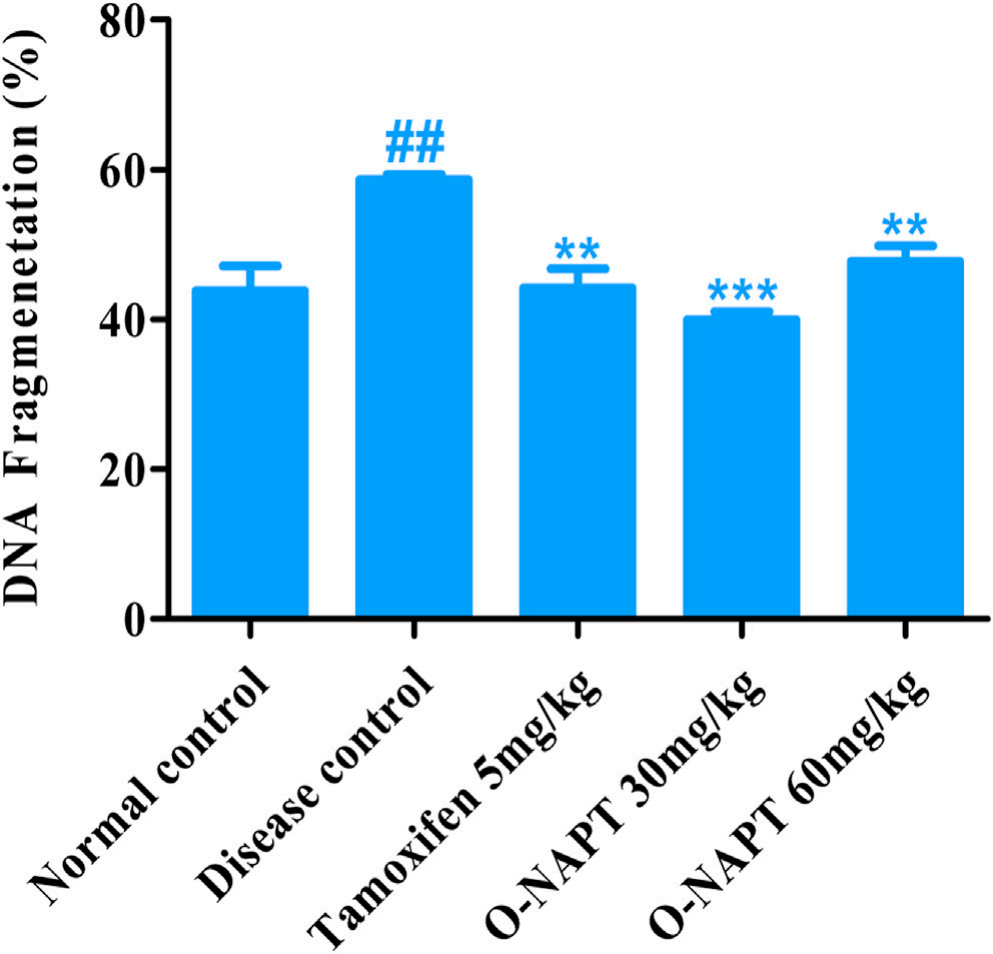
Effect of O-NPAT treatments on percentage of DNA fragmentation. Data are represented as mean ± SEM, *n* = 3, ##*p* < 0.01 and **p < 0.01, ***p < 0.001 as compared with normal control and diseased group, respectively.

The NMU (50 mg/kg)-treated animals exhibited a moderately significant increase in the DNA strand breakage, indicating cellular damage (58.66 ± 0.669%). O-NPAT (30 mg/kg) presented a highly significant reduction (*p* < 0.001) in percentage fragmentation in contrast to the disease control group, demonstrating the treatment efficacy (39.9 ± 1.037%). Meanwhile, tamoxifen (5 mg/kg) and O-NPAT (60 mg/kg) showed a moderately significant decrease (*p* < 0.01) in fragmented DNA nearly equivalent to that in the normal control group (Figure 8).

### Effect of O-NPAT Treatments on Hormonal Profile

**FIGURE 9:**
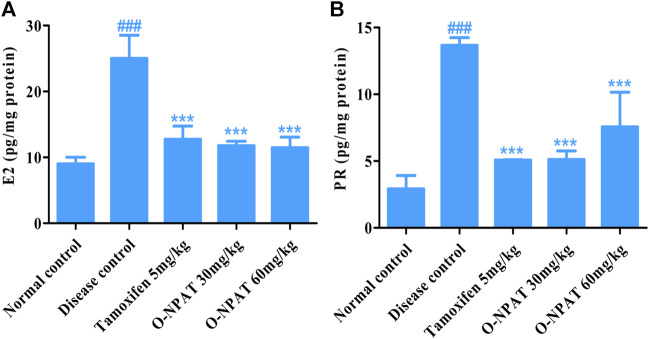
Effect of O-NPAT treatments on the hormonal profile in rats treated with NMU. **(A)** Estradiol (E2) levels. **(B)** Progesterone levels. Data are represented as mean ± SEM, *n* = 3, ###*p* < 0.001 and ***p < 0.001 as compared with normal control and diseased group, respectively.

Estradiol (E_2_) and progesterone are the significant components of normal mammary glands’ structural and functional integrity, increased levels of which are associated with disease condition. Therefore, the level of these hormones was monitored for all experimental group animals. The NMU (50 mg/kg)-treated animals showed a highly significant increase (*p* < 0.001) in the E_2_ level (25.03 ± 2.028 pg/mg). All treatment groups displayed dosage efficacy *via* a highly significant reduction (*p* < 0.001) in the E_2_ hormone as compared to the disease control group (Figure 9A), whereas for progesterone, the results showed tamoxifen (5 mg/kg) and O-NPAT at both doses of 30 and 60 mg/kg exhibiting a highly significant reduction (*p* < 0.001) in PR levels in contrast to the disease control group, the tumor characteristics (13.6 ± 0.332 pg/mg) of which are presented in Figure 9B.

### Effect of O-NPAT Treatments on Inflammatory Mediator Level

**FIGURE 10:**
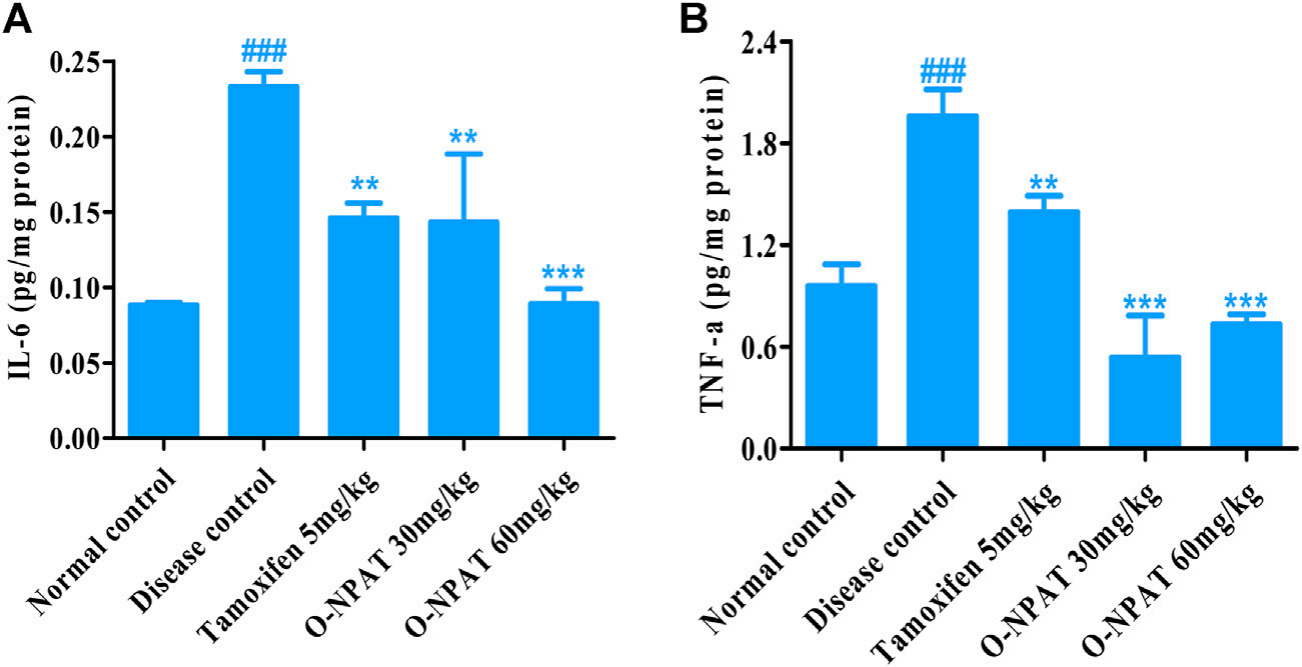
Effect of O-NPAT treatments on the inflammatory mediator level in rats treated with NMU. **(A)** Interleukin-6 (IL-6) level. **(B)** Tumor necrosis factor-alpha (TNF-α) level. Data are represented as mean ± SEM, *n* = 3, ###*p* < 0.001 and ***p < 0.001, **p < 0.01 as compared with normal control and diseased group, respectively.

The inflammatory cytokines including interleukins and tumor necrosis factor-alpha play a significant role in many disease conditions including cancer. Therefore, the level of these mediators was monitored in all experimental group animals. O-NPAT (60 mg/kg) exhibited a highly significant decrease (*p* < 0.001) in IL-6 levels, that is, parallel to the normal control group (0.143 ± 0.026 pg/mg) in comparison to the disease control group, which displayed a highly significant increase (*p* < 0.001) in interleukin-6 (0.233 ± 0.006 pg/mg), indicative of the disease features (Figure 10A).

The results for tumor necrosis factor-alpha showed O-NPAT treatments presenting a highly significant reduction (*p* < 0.001) in contrast to the disease control group, which showed highly significantly enhanced (*p* < 0.001) TNF-α levels in comparison to the normal control group (1.96 ± 0.091 pg/mg) (Figure 10B).

### Histopathological Analysis

**FIGURE 11:**
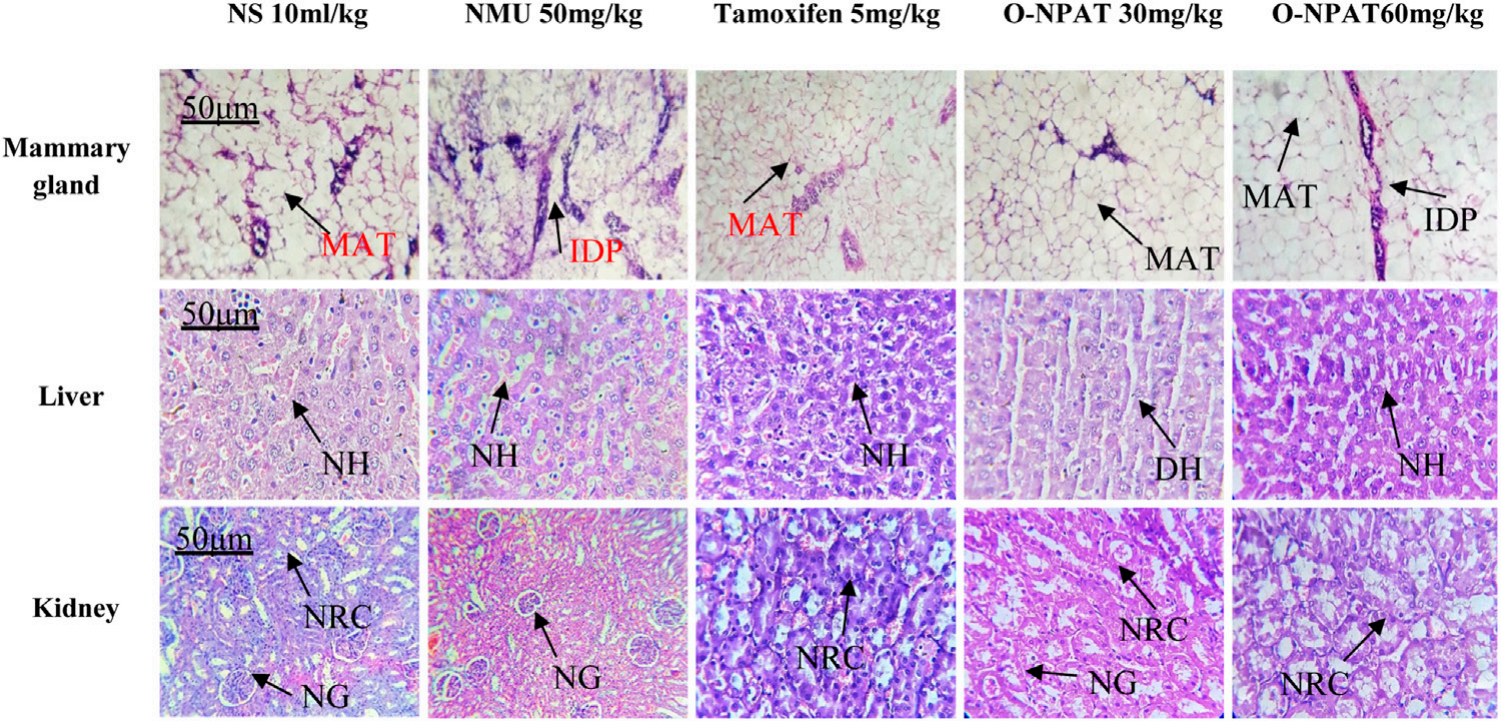
Effect of O-NPAT treatments on the mammary gland, liver, and kidney histoarchitecture against NMU-induced toxicity in female rats. MAT, mammary adipose tissues; IDP, intraductal proliferation; NH, normal hepatocytes; DH, ductal hyperplasia; NRC, normal renal cells; and NG, normal glomeruli.

Histopathological findings of all experimental group animals were compared for NMU-induced tissue abnormalities and O-NPAT treatment refinements. The normal control group showed the normal structure of mammary glands like profusion of normal epithelia, ducts, stroma, and mammary adipose tissues. The NMU (50 mg/kg)-treated disease model displayed neoplastic features with numerous intraductal proliferations (IDPs), which are the typical initial lesions of any mammary gland tumor. These animals also showed the characteristics of papillary carcinomas (a network of neoplastic epithelial aberrations), which are the most frequent form of NMU-induced tumors. Treatment with tamoxifen (5 mg/kg) showed an excess of normal stroma and epithelia, with a mild increase in periductal tissues. Treatment with O-NPAT at both doses of 30 and 60 mg/kg presented a mammary gland histoarchitecture comparable to that of the normal control group, with an abundance of normal stroma and normal adipose tissues alongside a few intraproliferated ducts (Figure 11).

#### Liver

Histological examination of the liver presented the normal control group with normal hepatocytes scattered around the central hepatic vein and normal peripheral features. The disease control group (NMU) showed normal tissue characteristics with insignificant ductal hyperplasia. The standard control rats showed adequate normal hepatic cells and normal vascular tissues. Treatment with O-NPAT at a dose of 30 mg/kg showed a normal tissue structure alongside a few vascular spaces, without any indications of liver necrosis. O-NPAT (60 mg/kg) also presented intact hepatocytes and a normal organ histoarchitecture (Figure 11).

#### Kidney

The kidney for the normal control group showed normal renal glomeruli and tubules of the cortical region lined by thin epithelia. The disease control group (NMU 50 mg/kg) also presented normal tissue characteristics without any indications of necrosis. Tamoxifen (5 mg/kg)-treated rats showed immature glomeruli with normal peripheral features. Treatment with O-NPAT (30 and 60 mg/kg) showed plentiful normal tissue features with the usual tubules and glomeruli alongside insignificant epithelial hyperplasia, where no cellular necrosis was seen (Figure 11).

## Discussion

A worldwide increase in breast cancer morbidity, adverse drug events, drug resistance, and cancer relapse has demanded the search for new breast cancer therapies. This purpose has led to the extensive studies of the novel 1,3,5-triazine class of medicinal agents, which has shown usefulness in anticipation of various cancer types including breast cancer (Yan et al., 2018). Most notably, the alkylating agent altretamine [2,4,6 Tris (dimethyl amino) 1,3,5-triazine] is currently being employed as an authorized second-line medication for ovarian cancer therapy (Keldsen et al., 2003). Monastrol derivatives and 1,2,4-triazines had proven activity against human breast cancer, as demonstrated by Krauth et al. (2010). Also, the fluoro and imidazolyl 1,3,5-triazines had potent antiproliferative action toward breast carcinoma, as shown by Singla et al. (2015). This study was designed to appraise the anticancer potential of a triazine-based compound—2,4,6 (O-nitrophenyl amino) 1,3,5- triazine (O-NPAT)—*via* computational modeling, *in vitro* cytotoxicity assay, and biochemical and histological analyses.

*In silico* modeling presented O-NPAT (binding energy = −7.1 kcal/mol) having good interaction at EGFR in comparison to tamoxifen (binding energy = −7.7 kcal/mol) but showing great deviation at ERα, ERβ, and PR. The docking simulations provided insight into the treatment compound’s ability to activate its target receptors. The cytotoxicity assay presented the 1C_50_ value to be 30 μm with a 15% inhibition of adenocarcinoma cells and helped design the safe and effective dosage for *in vivo* experimentation. O-NPAT at the dose of 30 and 60 mg/kg was evaluated for anticancer effect against N-Nitroso-N-methyl urea (NMU)–triggered mammary gland tumor in female rats. The decrease in the body weight of the animals during NMU treatment shows the morbid state and the induction of breast tumor associated with it. When compared for organ weight, the NMU (50 mg/kg)-treated rats presented the highest mammary gland weight (0.52 ± 0.051g), indicating the induction of breast carcinogenesis (Adefisan et al., 2019). The significant reduction (*p* < 0.05) in the organ weight of the treatment control animals gave us a clue with regards to the protective role of the dosage in tumor suppression. The results for LFTs and RFTs also displayed the O-NPAT capability to restore the hepatic and renal indices near to the normal control values and amplify the safety profile of the test compound (Table 2).

The association between NMU-provoked mammary gland carcinogenesis and oxidative stress is well-recognized (Valko et al., 2006). Cellular oxidative stress that is the result of excessive generation of reactive free radicals, or reduction in natural antioxidant defense mechanisms is the hallmark of several disease conditions including cancer (Fuchs-Tarlovsky, 2013). The antioxidant enzyme superoxide dismutase (SOD) performs the superoxide radical (O^2-^) dismutation into the less reactive hydrogen peroxide (H_2_O_2_) and molecular oxygen (O_2_), and the catalase (CAT) enzyme further decomposes H_2_O_2_ into oxygen and water and averts cellular damage from ROS (Nandi et al., 2019). Both these enzymes are important cellular defense participants, where SOD plays the foremost role of limiting the oxidative stress related to disease pathogenesis, while CAT performs the neutralization of cellular reactive species (Valko et al., 2006). The treatment with O-NPAT (30 and 60 mg/kg) presented a highly significant increase in SOD (*p* < 0.05) and CAT (*p* < 0.001) activity, respectively. Other antioxidant enzyme activities include nitric oxide (NO) and glutathione (GSH) estimation, where nitric oxide is an important signaling molecule and vasodilator and glutathione contributes to the immune system, regulates biochemical reactions, metabolizes drug toxins, and also serves as an antioxidant (Pompella et al., 2003). O-NPAT at both doses exhibited a moderately significant increase (*p* < 0.01) in nitric oxide and restored GSH levels in parallel to the normal control group. Levels of sulfhydryl compounds, which contribute to protein tertiary structure formation, were also found to be highly significantly improved (*p* < 0.001). The results demonstrated the O-NPAT anticancer effect against oxidative stress biomarkers where it attenuated the NMU-triggered diminution of defensive antioxidants and was able to restore biochemical enzyme levels in comparison to the disease control group. O-NPAT was also tested for disease biomarkers, where MDA is a highly reactive aldehyde formed of polyunsaturated fatty acid degradation or lipid peroxidation (Del Rio et al., 2005) and LDH is a by-product of tissue breakdown and also acts as a tumor marker (Girgis et al., 2014). Results showed the NMU (50 mg/kg)-treated animals with a substantial increase in disease biomarkers indicating mammary gland carcinogenesis. The noticeable downturn (*p* < 0.001) in lipid peroxidation and LDH in the treatment control animals shows the treatment competence in the eradication of lipid peroxidation and tissue injury. Overall, the test compound showed antitumor properties *via* upregulation of protective antioxidant enzyme levels in parallel to the normal control group and significant downfall in disease biomarkers and helped to cure the NMU-induced toxicity of mammary glands.

Besides biochemical parameters, DNA fragmentation or cell-free DNA is the hallmark of cellular apoptotic mechanisms (Sakkas et al., 2010). The results showed the disease control group with the highest percentage of DNA strand breakage (58.66 ± 0.669%) that evidenced the carcinogenic effect of NMU on genetic components. The treatment drug, O-NPAT (30 and 60 mg/kg), showed a highly significant (*p* < 0.001) and little significant (*p* < 0.05) reduction in percentage DNA breakdown, respectively. This reduction in fragmented DNA is a distinguishing feature of normal organ cells and establishes the O-NPAT treatment’s shielding effect against DNA strand disassembly and pronounced genomic stability with subsequent anticancer properties.

Hormones of the female body such as estrogen and progesterone are the most important elements, the increased level of which participates in cancer development, pathogenesis, and treatment complications by modulating cellular functions and metastasis (Segaloff, 2013). The ELISA quantitation of estradiol (E2) and progesterone showed these proteins to be strongly expressed (*p* < 0.001) in NMU (50 mg/kg)-treated animals, indicating the cancerous state of the mammary gland cells. The treatment with O-NPAT at both doses displayed a highly significantly reduced (*p* < 0.001) E_2_ and PR level and thus verified the subdue incidence of mammary gland tumor (Figure 9). O-NPAT acts on EGFR, which was found to be overexpressed in nearly 15–20% of ER+ tumors, where clinical trials have also shown the efficacy of adjuvant HER2 modulators along with tamoxifen for ER+ breast tumors (Cleator et al., 2009). In the current study, the highly significantly decreased estradiol and progesterone levels can thus be interlinked with the activity of O-NPAT while acting on EGFR. Besides antioxidant enzymes and hormonal activity, the role of inflammatory cytokines (IL-6 and TNF-α) in disease pathology cannot be overlooked. They participate in tissue inflammation, disrupted cellular signaling, and overall poor prognosis (Tripsianis et al., 2014). O-NPAT was found to have a highly significant (*p* < 0.001) reduction in IL-6 and TNF-α levels at both doses, which further strengthens the hypothesis of O-NPAT attenuation of mammary gland carcinogenesis.

The histopathological findings correlated with those of Adefisan et al. (2019), where the disease control animals presented the abundance of ductal proliferations, which developed into invasive ductal carcinoma. Tamoxifen (5 mg/kg) and O-NPAT at both doses ameliorated the tumor expression with plentiful normal stroma, ducts, and adipose tissues and verified the O-NPAT protective effect on organs’ anatomical structure and physiological functioning, as shown in Figure 11.

Overall, the test compound showed a reduction in the breast tumor burden, where it establishes its curative effect against multiple disease variables. The cytotoxicity of the drug was analyzed on an array of variables such as liver and renal function, protein levels, antioxidant enzymes, DNA fragmentation, and hormonal and interleukin levels. O-NPAT was able to reinstate the protective enzymatic activity with the decrease in the hormonal level at the dose level of 30 and 60 mg/kg, where results were found to be comparable to those of the normal control group and the standard control group (tamoxifen 5 mg/kg). All the results are indicative of the treatment compound efficacy to counteract hormone-positive breast malignancy.

## Conclusion

O-NPAT demonstrated anticancer activity *via in silico* modeling, *in vitro* cytotoxicity, and *in vivo* experimentation. O-NPAT treatments significantly restored the level of antioxidant enzymes and showed a decline in lipid peroxidation and fragmented DNA and inflammatory mediators. A substantial decrease in the expression of estradiol and progesterone also showed the annihilation of breast tumor characteristics. Histopathological analysis presented the treatment competence to restore the normal tissue features. This significant reduction in disease hallmarks is the showcase of the O-NPAT antitumor capabilities to counteract breast malignancy and presents its possibility of exploitation for future studies.

## Data Availability

The raw data supporting the conclusions of this article will be made available by the authors, without undue reservation.
